# Supplementary material for: COX7A1-mediated mitochondrial dysfunction can induce ferroptosis in endometrial cancer cells
Source: PLoS One. 2026 Feb 23;21(2):e0342333. doi: 10.1371/journal.pone.0342333 (PMC12928431; doi:10.1371/journal.pone.0342333)

Figure3A COX7A1

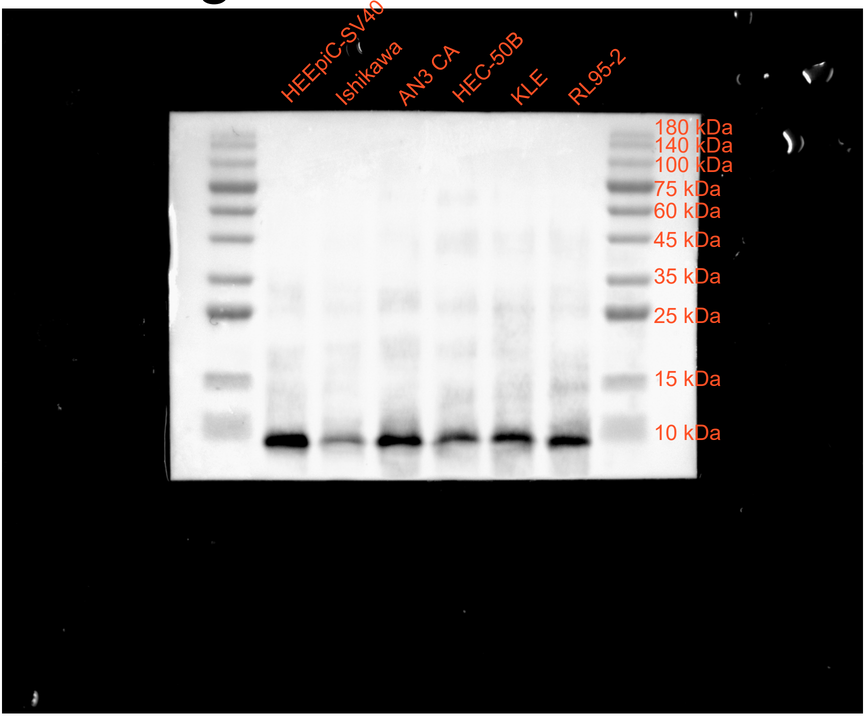

Figure3A GAPDH

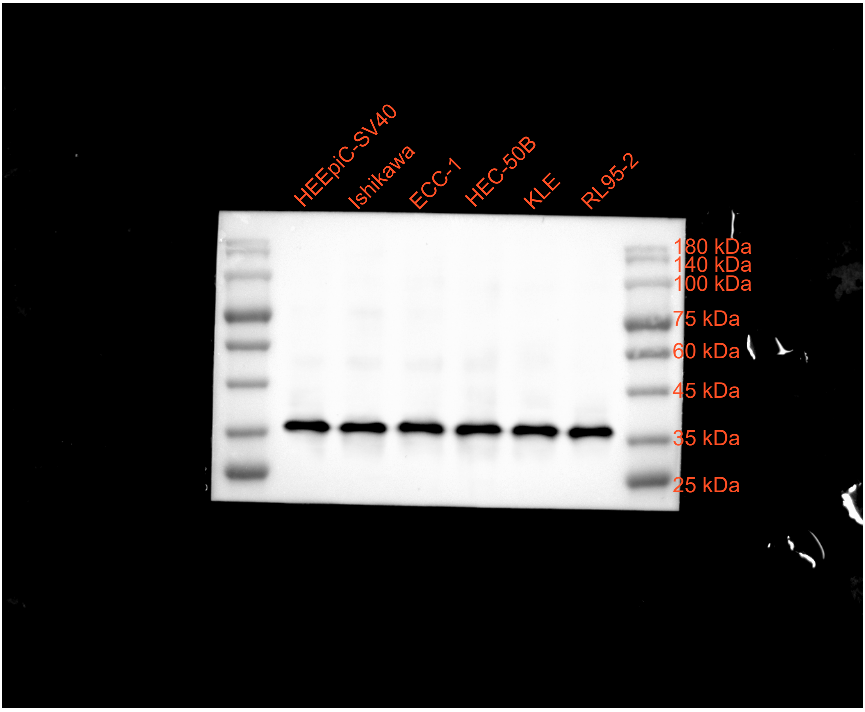

Figure6C COX7A1

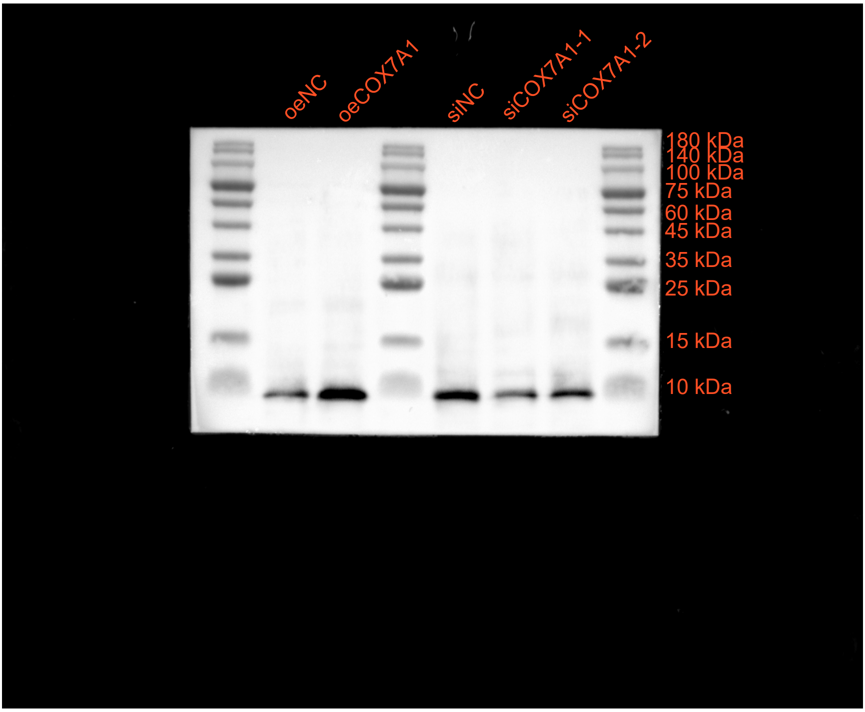

Figure6C GPX4

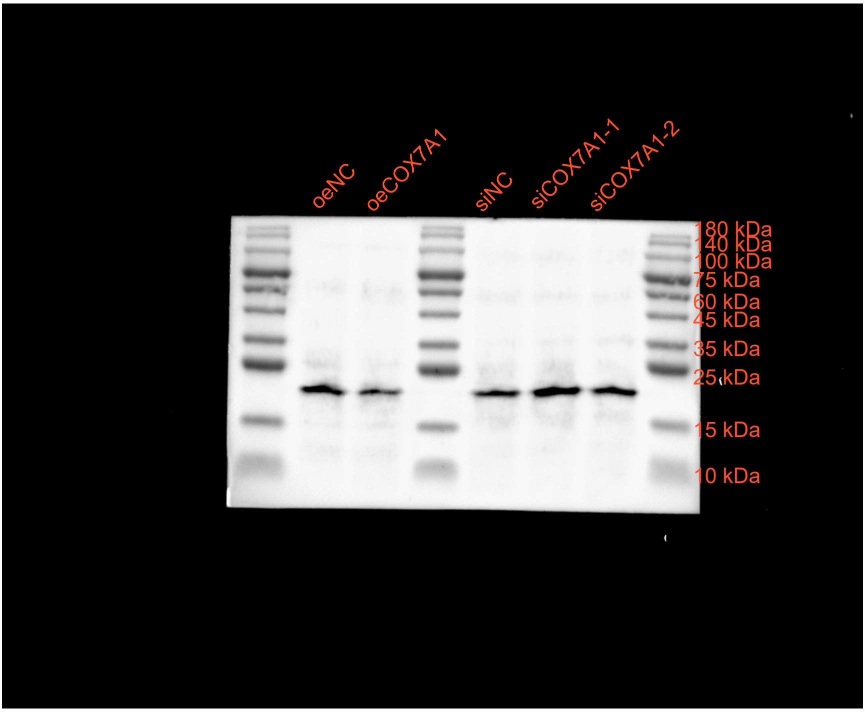

Figure6C ASCL4

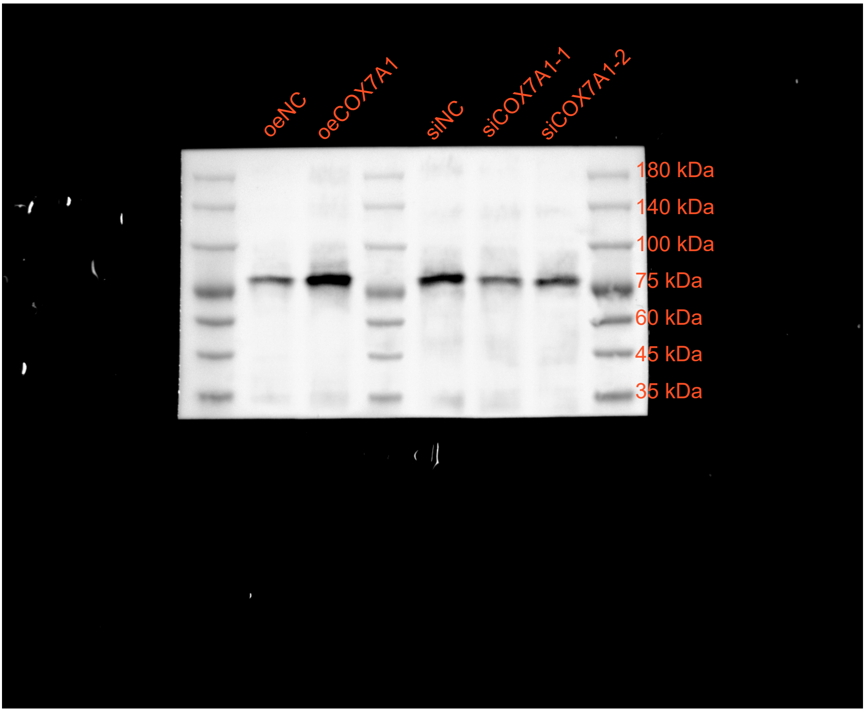

Figure6C SCL7A11

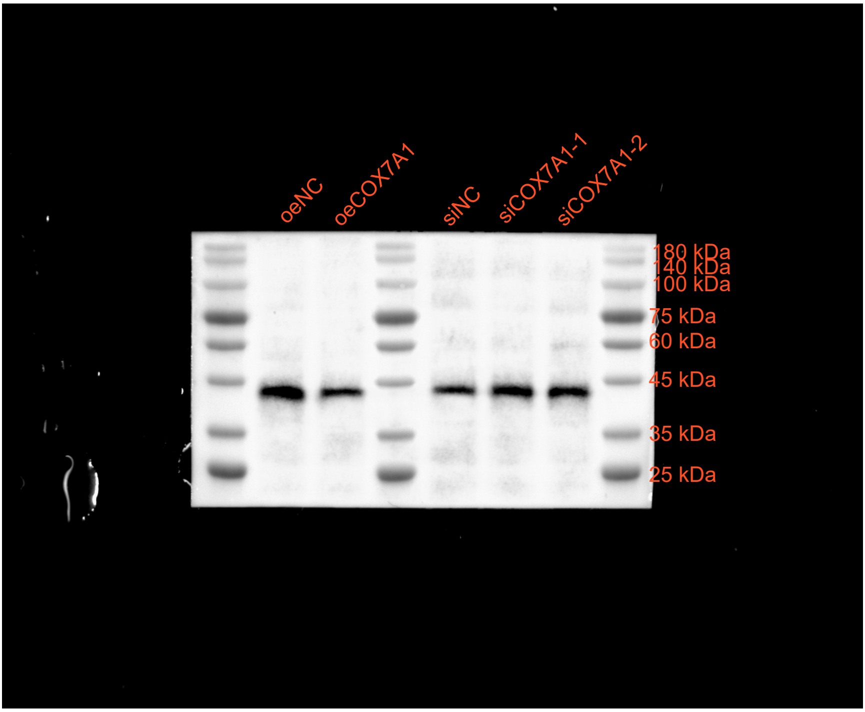

Figure6C GAPDH

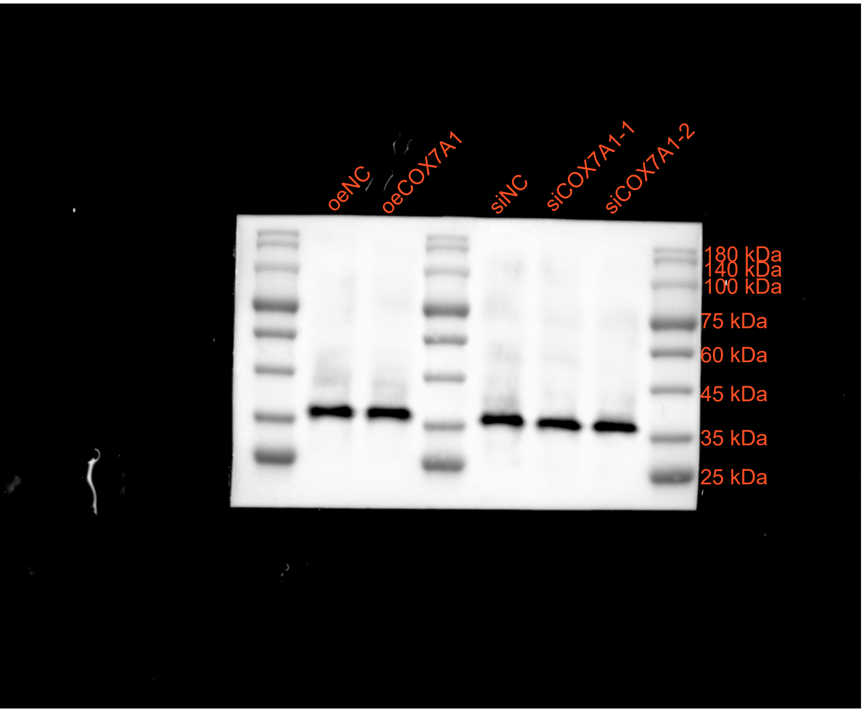

Figure7B VDAC1

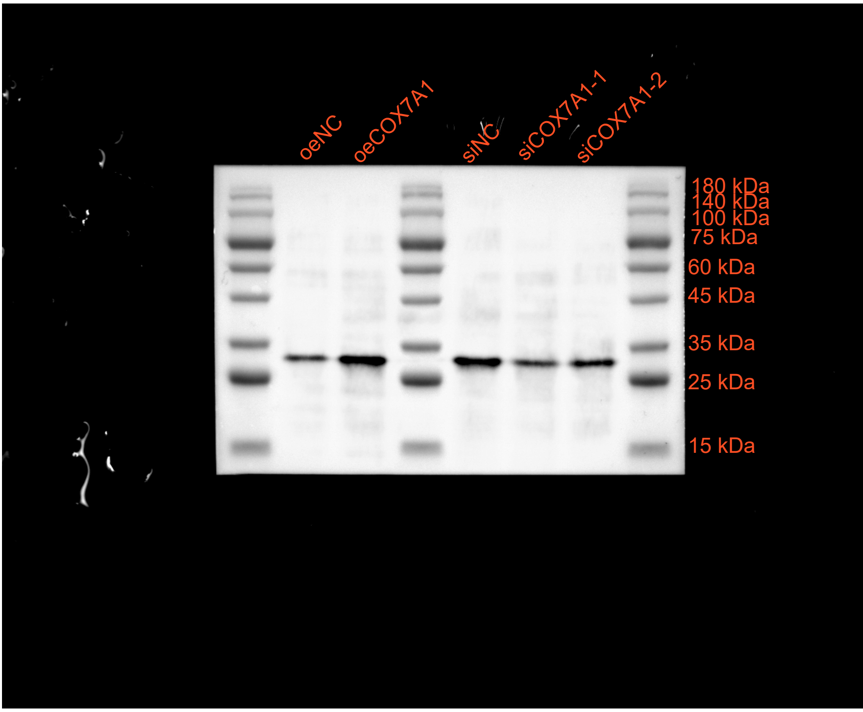

Figure7B Cytochrome C

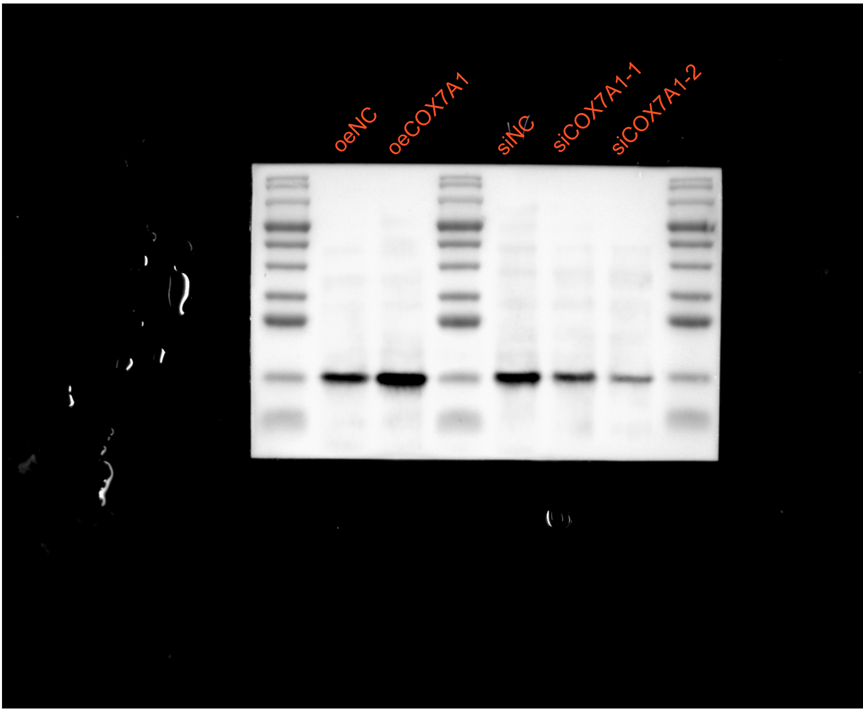

Figure7B ATP5A1

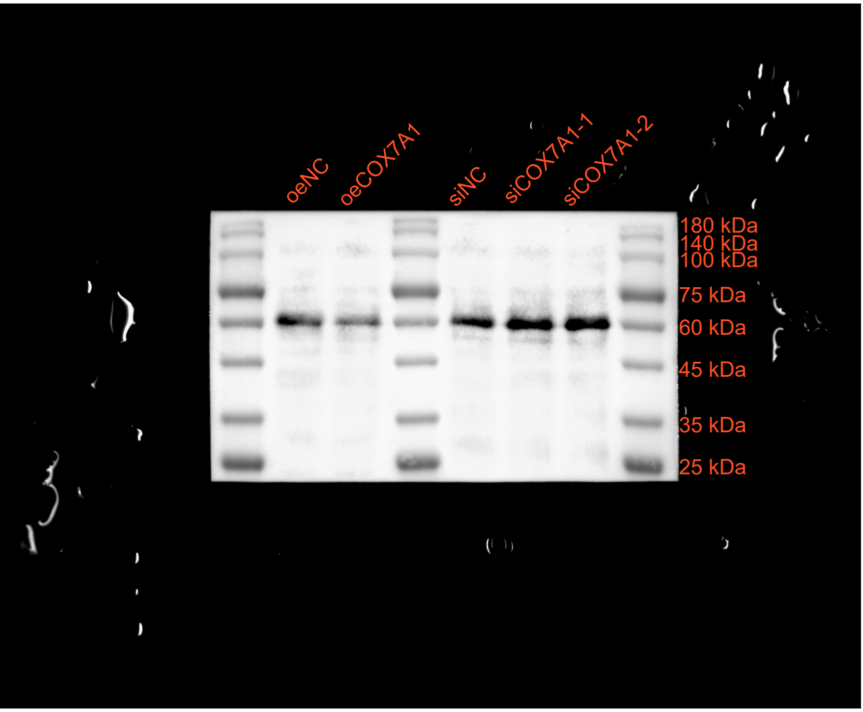

Figure7B OPA1

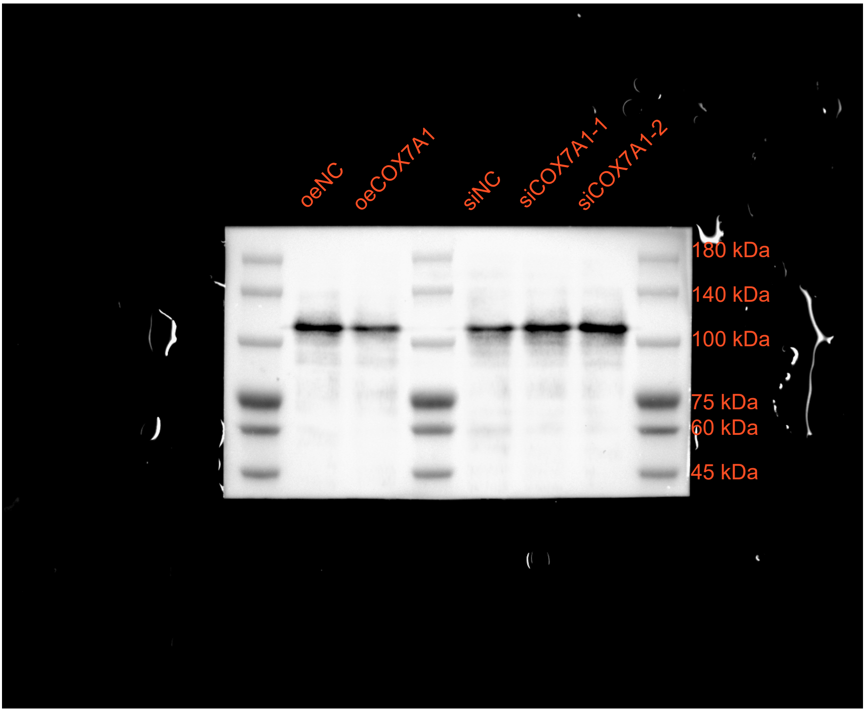

Figure7B GAPDH

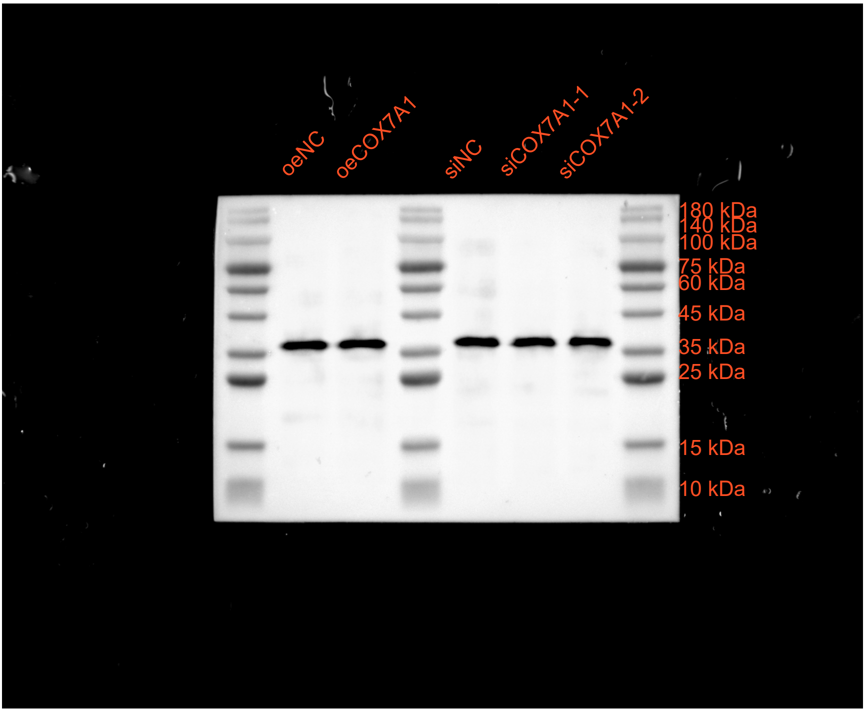

Supplement: S1 File — (PDF) [file pone.0342333.s001.pdf]
