# Supplementary material for: COX7A1-mediated mitochondrial dysfunction can induce ferroptosis in endometrial cancer cells
Source: PLoS One. 2026 Feb 23;21(2):e0342333. doi: 10.1371/journal.pone.0342333 (PMC12928431; doi:10.1371/journal.pone.0342333)
Supplement: S2 File — (PDF) [file pone.0342333.s002.pdf]

Fig. 3A qPCR

qPCR Analysis Report							
Gene	Group	GAPDH	Target Gene	ΔCt	—ΔΔCt	Relative Expression	Relative Mean Value
COX7A1		20.75324	23.77080217	3.017557	-0.038873605	0.973414652	
	HEEpiC-SV40	20.65809	23.69942084	3.041333	-0.062649479	0.957504068	1.00127
		20.52346	23.40062026	2.877161	0.101523083	1.072905554	
		21.30511	26.73058994	5.425478	-2.446794297	0.183417818	
	Ishikawa	20.83802	26.34702418	5.509003	-2.530319327	0.173100365	0.16615
		20.80724	26.60278113	5.795543	-2.816858988	0.141919134	
		20.36547	23.82273849	3.457265	-0.478581173	0.717683087	
	ECC-1	20.45774	23.8994195	3.441682	-0.462997916	0.725477152	0.79093
		20.75945	23.84340809	3.08396	-0.105276283	0.929626898	
		20.37133	24.32273849	3.951413	-0.972729114	0.509541263	
	HEC-50B	20.54468	24.9994195	4.45474	-1.476056123	0.359470149	0.46087
		20.30346	24.24340809	3.939951	-0.961267569	0.513605455	
		21.56276	25.82393308	4.261171	-1.282487197	0.411086188	
	KLE	21.62295	25.96094638	4.338	-1.359315825	0.389767087	0.39773
		21.56059	25.88914921	4.32856	-1.34987629	0.392325689	
		21.81943	25.86998924	4.050556	-1.071871819	0.475701402	
	RL95-2	21.77701	25.54097866	3.763973	-0.78528883	0.580235782	0.54026
		21.97911	25.78183977	3.802734	-0.824050212	0.564853948	

Fig. 3A Western blot

HEEpiC-SV40	1.070517	0.978269	0.859099
Ishikawa	0.370871	0.471358	0.577776
ECC-1	0.936796	0.799307	0.747095
HEC-50B	0.627266	0.506072	0.50909
KLE	0.671416	0.63421	0.697814
RL95-2	0.76025	0.742743	0.722402

Fig. 3B

qPCR Analysis Report							
Gene	Group	GAPDH	Target Gene	ΔCt	—ΔΔCt	Relative Expression	Relative Mean Value
COX7A1		20.5492	26.24820714	5.699008621	-0.122777639	0.918417706	
	oeNC	20.85409	26.36535093	5.511261623	0.06496936	1.046062723	1.00179
		20.69412	26.21254502	5.518422704	0.057808278	1.040883264	
		20.58221	23.40806994	2.825864655	2.750366328	6.728879695	
	oeCOX7A1	20.7556	23.58934587	2.833741233	2.74248975	6.692242622	6.28325
		20.42173	23.55737658	3.135643243	2.44058774	5.428628428	
		20.93992	26.77452303	5.834605213	-0.01192553	0.991767923	
	siNC	20.95438	26.77122902	5.816845466	0.005834216	1.004052158	1.00002
		20.57247	26.38905963	5.816588368	0.006091315	1.004231103	
		20.96435	28.84854383	7.884197022	-2.06151734	0.239563938	
	siCOX7A1-1	20.7978	28.53109261	7.733287723	-1.910608041	0.265980421	0.25226
		20.96419	28.77982104	7.815626972	-1.99294729	0.251225134	
		21.08229	28.54162165	7.4593303	-1.636650618	0.321602245	
	siCOX7A1-2	20.63865	28.9096886	8.271034527	-2.448354845	0.183219524	0.29035
		21.10485	28.37676676	7.271920916	-1.449241233	0.366213979	

Fig. 3C

oeNC	0.956918	0.925544	1.117538
oeCOX7A1	2.751709	2.882697	2.731405
siNC	0.953098	1.084172	0.962731
siCOX7A1-1	0.274063	0.343732	0.300071
siCOX7A1-2	0.377902	0.406339	0.313798

Fig. 4A

	oenc	oeCOX7A1	siNC	siCOX7A1-1	siCOX7A1-2
DAPI	52066	51405	51718	50917	54986
DAPI	54494	53230	53420	50475	52123
DAPI	50163	51353	50287	50770	54017
EDU	18223	7711	12930	22913	18695
EDU	16348	10646	14958	20190	18243
EDU	17557	8730	15086	19293	22687
EDU positive rate	oenc	oeCOX7A1	siNC	siCOX7A1-1	siCOX7A1-2
	35%	15%	25%	45%	34%
	30%	20%	28%	40%	35%
	35%	17%	30%	38%	42%

Fig. 4B

	0h											
siNC	0.455	0.432	0.487	0.502	0.428	0.344	0.331	0.323	0.333	0.328	0.342	0.329
siCOX7A1-1	0.555	0.501	0.422	0.385	0.355	0.339	0.337	0.348	0.321	0.317	0.336	0.334
siCOX7A1-2	0.452	0.552	0.4521	0.552	0.481	0.321	0.347	0.3	0.314	0.335	0.304	0.335
oeNC	0.523	0.551	0.542	0.425	0.435	0.318	0.336	0.332	0.331	0.326	0.318	0.324
oeCOX7A1	0.521	0.582	0.433	0.485	0.417	0.308	0.347	0.329	0.341	0.336	0.308	0.311
	0.323	0.32	0.326	0.314	0.302	0.32	0.309	0.333	0.35	0.35	0.315	0.34
	0.304	0.321	0.334	0.336	0.345	0.342	0.338	0.338	0.342	0.324	0.311	0.306
	0.31	0.35	0.307	0.314	0.344	0.325	0.341	0.34	0.35	0.311	0.343	0.349
24h												
siNC	0.578	0.552	0.501	0.621	0.587	0.309	0.309	0.305	0.327	0.344	0.328	0.324
siCOX7A1-1	0.624	0.655	0.621	0.645	0.588	0.343	0.333	0.311	0.316	0.341	0.308	0.33
siCOX7A1-2	0.521	0.552	0.458	0.621	0.578	0.35	0.329	0.302	0.319	0.332	0.306	0.316
oeNC	0.658	0.705	0.627	0.685	0.624	0.329	0.334	0.349	0.349	0.325	0.303	0.34
oeCOX7A1	0.584	0.551	0.532	0.482	0.562	0.333	0.302	0.347	0.303	0.303	0.331	0.306
	0.305	0.34	0.329	0.313	0.308	0.34	0.31	0.3	0.335	0.343	0.337	0.337
	0.305	0.326	0.334	0.333	0.349	0.307	0.329	0.307	0.335	0.323	0.344	0.34
	0.323	0.341	0.34	0.319	0.33	0.318	0.318	0.313	0.333	0.327	0.309	0.331
	48h											
siNC	0.785	0.821	0.695	0.755	0.745	0.307	0.308	0.331	0.345	0.347	0.324	0.312
siCOX7A1-1	0.958	0.887	0.902	1.021	0.888	0.344	0.304	0.318	0.312	0.325	0.339	0.336
siCOX7A1-2	0.958	1.025	0.998	0.951	0.921	0.346	0.323	0.328	0.341	0.325	0.31	0.318
oeNC	0.845	0.955	0.881	0.843	0.837	0.338	0.328	0.344	0.341	0.32	0.319	0.309
oeCOX7A1	0.652	0.687	0.758	0.602	0.624	0.344	0.327	0.342	0.329	0.338	0.338	0.327
	0.316	0.34	0.347	0.348	0.304	0.314	0.309	0.302	0.337	0.332	0.321	0.302
	0.334	0.346	0.335	0.317	0.319	0.312	0.33	0.306	0.344	0.319	0.321	0.307
	0.304	0.343	0.3	0.333	0.32	0.326	0.321	0.329	0.348	0.333	0.324	0.336
72h												
siNC	0.998	1.085	1.174	1.245	1.234	0.349	0.308	0.34	0.327	0.303	0.339	0.335
siCOX7A1-1	1.452	1.421	1.32	1.254	1.488	0.317	0.328	0.303	0.315	0.339	0.34	0.327
siCOX7A1-2	1.587	1.652	1.254	1.325	1.452	0.335	0.342	0.3	0.327	0.325	0.307	0.344
oeNC	1.246	1.345	1.442	1.423	1.325	0.338	0.306	0.341	0.34	0.317	0.349	0.34
oeCOX7A1	0.852	0.877	0.785	0.921	0.798	0.31	0.315	0.338	0.328	0.312	0.347	0.33
	0.335	0.343	0.329	0.331	0.31	0.342	0.314	0.341	0.328	0.308	0.319	0.343
	0.3	0.326	0.311	0.341	0.318	0.337	0.312	0.348	0.348	0.342	0.332	0.314
	0.34	0.345	0.31	0.329	0.342	0.326	0.302	0.35	0.319	0.32	0.348	0.322

	siNC					siCOX7A1-1					siCOX7A1-2				
0 h	0.455	0.432	0.487	0.502	0.428	0.555	0.501	0.422	0.385	0.355	0.452	0.552	0.4521	0.552	0.481
24 h	0.578	0.552	0.501	0.621	0.587	0.624	0.655	0.621	0.645	0.588	0.521	0.552	0.458	0.621	0.578
48 h	0.785	0.821	0.695	0.755	0.745	0.958	0.887	0.902	1.021	0.888	0.958	1.025	0.998	0.951	0.921
72 h	0.998	1.085	1.174	1.245	1.234	1.452	1.421	1.32	1.254	1.488	1.587	1.652	1.254	1.325	1.452

	oeNC					oeCOX7A1				
0 h	0.523	0.551	0.542	0.425	0.435	0.521	0.582	0.433	0.485	0.417
24 h	0.658	0.705	0.627	0.685	0.624	0.584	0.551	0.532	0.482	0.562
48 h	0.845	0.955	0.881	0.843	0.837	0.652	0.687	0.758	0.602	0.624
72 h	1.246	1.345	1.442	1.423	1.325	0.852	0.877	0.785	0.921	0.798

Fig. 5A

siNC	1	0.0879	0.08	0.054	8.44	7.50	1.13	1.24
	2	0.0902		0.056	8.80		1.17	
	3	0.1021		0.068	10.66		1.42	
siCOX7A1-1	4	0.0686		0.035	5.42		0.72	0.71
	5	0.0712		0.037	5.83		0.78	
	6	0.0635		0.030	4.63		0.62	
siCOX7A1-2	1	0.0587	0.07	0.025	3.88		0.52	0.69
	2	0.0775		0.044	6.81		0.91	
	3	0.0652		0.031	4.89		0.65	
oeNC	4	0.0884		0.055	8.52		1.14	0.93
	5	0.0784		0.045	6.95		0.93	
	6	0.0685		0.035	5.41		0.72	
oeCOX7A1	1	0.1546	0.14	0.121	18.86		2.51	2.17
	2	0.1245		0.091	14.16		1.89	
	3	0.1348		0.101	15.77		2.10	

Fig. 5B

siNC	1	0.0897	3.25	0.176	0.06	0.32	3.18027	0.98	1.02
	2	0.0998	3.15		0.07	0.38	3.75283	1.19	
	3	0.0854	3.25		0.05	0.29	2.93651	0.90	
siCOX7A1-1	1	0.0521	3.25		0.02	0.10	1.04875	0.32	0.39
	2	0.0558	3.45		0.02	0.13	1.2585	0.36	
	3	0.0621	3.25		0.03	0.16	1.61565	0.50	
siCOX7A1-2	1	0.0558	3.21		0.02	0.13	1.2585	0.39	0.46
	2	0.0587	3.25		0.03	0.14	1.4229	0.44	
	3	0.0654	3.21		0.03	0.18	1.80272	0.56	
oeNC	1	0.0887	3.15		0.06	0.31	3.12358	0.99	1.10
	2	0.1025	3.21		0.07	0.39	3.9059	1.22	
	3	0.0987	3.35		0.07	0.37	3.69048	1.10	
oeCOX7A1	1	0.2214	3.25		0.19	1.06	10.6463	3.28	3.59
	2	0.2546	3.15		0.22	1.25	12.5283	3.98	
	3	0.2345	3.23		0.20	1.14	11.3889	3.53	

Fig. 5C

	T-GSH（μmol/L）			GSSG（μmol/L）			GSH/GSSH
oeNC	11.66810717		oeNC	4.511278195	oeNC	1	2.586430424
	13.87208297			4.887218045		2	2.838441593
	12.83491789			3.383458647		3	3.793431288
oeCOX7A1	9.982713915		oeCOX7A1	6.337271751	oeCOX7A1	1	1.575238416
	5.834053587			6.015037594		2	0.969911409
	6.611927398			7.975295381		3	0.829051099
siNC	8.945548833		siNC	4.806659506	siNC	1	1.861073958
	10.37165082			5.155746509		2	2.011668107
	11.79775281			5.665950591		3	2.082219501
siCOX7A1-1	18.0207433		siCOX7A1-1	2.363050483	siCOX7A1-1	1	7.626050915
	14.13137424			3.249194415		2	4.349193197
	14.39066551			2.792696026		3	5.152965228
siCOX7A1-2	17.37251513		siCOX7A1-2	3.974221267	siCOX7A1-2	1	4.371300427
	15.68712187			3.759398496		2	4.172774417
	16.07605877			3.114930183		3	5.160969213

Fig. 6A

Mean ROS FITC-A	oenc	oecox7a1		sinc	sicox7a1-1	sicox7a-2
1	5889.4	12519.7		6556.4	3480.5	2810
2	5866.2	12127.5		6605.5	3432.8	2512
3	6133.7	11726.2		6512.9	3404.4	2434.5

Fig. 6B

	oeNC	oeCOX7A1	siNC	siCOX7A1-1	siCOX7A1-2
Mean area per	132531	149396	143084	142566	145226
mitochondria (Folds of control)	140726	142036	137044	148668	146697
	134712	138590	149009	148307	135869
	139158	82168	145946	152546	152487
	144948	85222	138414	160561	158433
	123935	73453	144539	163138	149456
	oeNC	oeCOX7A1	siNC	siCOX7A1-1	siCOX7A1-2
	1.05	0.55	1.02	1.07	1.05
	1.03	0.60	1.01	1.08	1.08
	0.92	0.53	0.97	1.10	1.10
Damaged mitochondria	58	55	56	55	57
	53	54	53	59	54
	60	53	50	59	55
	3	32	4	3	3
	4	35	3	3	3
	5	37	4	3	3
	5.63	57.385	7.85	4.88	5.72
	7.25	65.25	6.55	5.01	6.21
	8.21	70.46	7.02	5.41	5.12

Fig. 6C

Duplicate well 1	COX7A1	GPX4	ACSL4	SLC7A11
oeNC	0.960143	0.929089	0.9806	0.984592287
oe	1.868258	0.655148	2.172366	0.630407636
siNC	1.077887	1.117188	0.910054	0.93249185
si-1	0.389249	1.760031	0.61033	1.398433231
si-2	0.621728	0.855608	0.462966	1.247451441
Duplicate well 2	COX7A1	GPX4	ACSL4	SLC7A11
oeNC	1.141006	0.963907	0.899897	1.08378097
oe	2.018576	0.633212	2.275057	0.702991989
siNC	1.023949	0.970603	1.061593	1.012415771
si-1	0.298693	1.512738	0.712824	1.615591049
si-2	0.579982	1.346769	0.799537	1.1870255
Duplicate well 3	COX7A1	GPX4	ACSL4	SLC7A11
oeNC	0.89885	1.107004	1.119503	0.931626743
oe	1.7473	0.748673	2.346396	0.694701786
siNC	0.898164	0.912209	1.028353	1.055092379
si-1	0.315132	1.510785	0.688322	1.410359835
si-2	0.483985	1.340181	0.587242	1.454194822

Fig. 7A

	oeNC	oeCOX7A1	siNC	siCOX7A1-1	siCOX7A1-2
DAPI	45359	46287	45177	49379	48020
	46979	48479	49136	48726	48677
	46333	47003	47631	46214	46824
JC-1(R)	35264	25462	33254	38214	37562
	36521	28462	35462	35462	35462
	33254	23654	34526	36441	36542
JC-1(G)	41487	48965	31670	26723	30789
	34782	63249	38546	28602	30837
	30231	67583	33520	27001	28548
JC-1(R)/JC-1(G	oeNC	oeCOX7A1	siNC	siCOX7A1-1	siCOX7A1-2
	0.85	0.52	1.05	1.43	1.22
	1.05	0.45	0.92	1.24	1.15
	1.10	0.35	1.03	1.35	1.28

Fig. 7B

	VDAC1	VDAC1	VDAC1	Cytochrome C	Cytochrome C	Cytochrome C	ATP5A1	ATP5A1	ATP5A1	OPA1	OPA1	OPA1
oeNC	0.965772	1.076051	0.958177	0.982784906	1.024894997	0.992320096	1.058977	1.059203	0.88182	1.073003	0.963552	0.963445
oe	1.363514	1.416503	1.280606	1.377652014	1.340038392	1.120332511	0.701535	0.601676	0.545427	0.708983	0.777846	0.765837
siNC	0.9785	1.074545	0.946955	1.011799927	1.029816201	0.958383872	1.155851	0.983784	0.860364	0.897604	1.049754	1.052642
si-1	0.53552	0.475376	0.372397	0.765122286	0.687886749	0.55178525	1.725395	1.413955	1.30816	1.439746	1.494251	1.285211
si-2	0.640614	0.57237	0.642156	0.458518636	0.365351245	0.44434438	1.525275	1.632167	1.972064	1.590177	1.431172	1.797655
