# Supplementary material for: COX7A1-mediated mitochondrial dysfunction can induce ferroptosis in endometrial cancer cells
Source: PLoS One. 2026 Feb 23;21(2):e0342333. doi: 10.1371/journal.pone.0342333 (PMC12928431; doi:10.1371/journal.pone.0342333)
Supplement: Attachment — Submitted filename: Response to reviewers.pdf [file pone.0342333.s005.pdf]

Journal Requirements:

When submitting your revision, we need you to address these additional requirements.

1. Please ensure that your manuscript meets PLOS ONE's style requirements, including those for file naming. The PLOS ONE style templates can be found at https://journals.plos.org/plosone/s/file?id=wjVg/PLOSOne_formatting_sample_main_body.pdf and https://journals.plos.org/plosone/s/file?id=ba62/PLOSOne_formatting_sample_title_authors_affiliations.pdf

Response: We have edited our manuscript strictly in accordance with the format requirements to ensure it meets the requirements of your journal.

2. Please note that PLOS One has specific guidelines on code sharing for submissions in which author-generated code underpins the findings in the manuscript. In these cases, we expect all author-generated code to be made available without restrictions upon publication of the work. Please review our guidelines at https://journals.plos.org/plosone/s/materials-and-software-sharing#loc-sharing-code and ensure that your code is shared in a way that follows best practice and facilitates reproducibility and reuse.

Response: We have uploaded all the original R codes involved in the bioinformatics research of this study to the online system for inspection (https://figshare.com/s/5678c40e643bf55f363e).

3. PLOS ONE now requires that authors provide the original uncropped and unadjusted images underlying all blot or gel results reported in a submission's figures or Supporting Information files. This policy and the journal's other requirements for blot/gel reporting and figure preparation are described in detail at https://journals.plos.org/plosone/s/figures#loc-blot-and-gel-reporting-requirements and https://journals.plos.org/plosone/s/figures#loc-preparing-figures-from-image-files. When you submit your revised manuscript, please ensure that your figures adhere fully to these guidelines and provide the original underlying images for all blot or gel data reported in your submission. See the following link for instructions on providing

the original image data: https://journals.plos.org/plosone/s/figures#loc-original-images-for-blots-and-gels.

In your cover letter, please note whether your blot/gel image data are in Supporting Information or posted at a public data repository, provide the repository URL if relevant, and provide specific details as to which raw blot/gel images, if any, are not available. Email us at plosone@plos.org if you have any questions.

Response: We have uploaded all the original Western blot bands to the online database (https://figshare.com/s/5678c40e643bf55f363e).

4. We note that you have indicated that there are restrictions to data sharing for this study. PLOS only allows data to be available upon request if there are legal or ethical restrictions on sharing data publicly. For more information on unacceptable data access restrictions, please see http://journals.plos.org/plosone/s/data-availability#loc-unacceptable-data-access-restrictions.

Before we proceed with your manuscript, please address the following prompts:

a) If there are ethical or legal restrictions on sharing a de-identified data set, please explain them in detail (e.g., data contain potentially identifying or sensitive patient information, data are owned by a third-party organization, etc.) and who has imposed them (e.g., a Research Ethics Committee or Institutional Review Board, etc.). Please also provide contact information for a data access committee, ethics committee, or other institutional body to which data requests may be sent.

b) If there are no restrictions, please upload the minimal anonymized data set necessary to replicate your study findings to a stable, public repository and provide us with the relevant URLs, DOIs, or accession numbers. For a list of recommended repositories, please see

https://journals.plos.org/plosone/s/recommended-repositories. You also have the option of uploading the data as Supporting Information files, but we would recommend depositing data directly to a data repository if possible.

We will update your Data Availability statement on your behalf to reflect the information you provide.

In the online submission form, you indicated that the dataset associated with this study can be obtained from the lead researcher if a justified request is made.

All PLOS journals now require all data underlying the findings described in their manuscript to be freely available to other researchers, either 1. In a public repository, Within the manuscript itself, or 3. Uploaded as supplementary information.

This policy applies to all data except where public deposition would breach compliance with the protocol approved by your research ethics board. If your data cannot be made publicly available for ethical or legal reasons (e.g., public availability would compromise patient privacy), please explain your reasons on resubmission and your exemption request will be escalated for approval.

Response: We have updated the data availability statement and stored the original data of this study on the online website (https://figshare.com/s/5678c40e643bf55f363e).

6. Please include captions for your Supporting Information files at the end of your manuscript, and update any in-text citations to match accordingly. Please see our Supporting Information guidelines for more information: http://journals.plos.org/plosone/s/supporting-information.

Response: We have added the "Supporting Information" at the end of the article.

7. If the reviewer comments include a recommendation to cite specific previously published works, please review and evaluate these publications to determine whether they are relevant and should be cited. There is no requirement to cite these works unless the editor has indicated otherwise.

Response: We have read the literatures recommended by the reviewers, which are highly relevant to our research. Therefore, we have cited these literatures as required by the reviewers. Among them, one has been cited before, one is newly added, and for another one, due to incomplete information provided by the reviewers, we haven't been able to locate the corresponding literature.

[Note: HTML markup is below. Please do not edit.]

Reviewers' comments:

Reviewer's Responses to Questions

Comments to the Author

1. Is the manuscript technically sound, and do the data support the conclusions?

The manuscript must describe a technically sound piece of scientific research with data that supports the conclusions. Experiments must have been conducted rigorously, with appropriate controls, replication, and sample sizes. The conclusions must be drawn appropriately based on the data presented.

Reviewer #1: Partly

Reviewer #2: Yes

---

2. Has the statistical analysis been performed appropriately and rigorously?

Reviewer #1: Yes

Reviewer #2: Yes

---

3. Have the authors made all data underlying the findings in their manuscript fully available?

The PLOS Data policy requires authors to make all data underlying the findings described in their manuscript fully available without restriction, with rare exception (please refer to the Data Availability Statement in the manuscript PDF file). The data should be provided as part of the manuscript or its supporting information, or deposited to a public repository. For example, in addition to summary statistics, the data points behind means, medians and variance measures should be available. If there are restrictions on publicly sharing data—e.g. participant privacy or use of data from a third party—those must be specified.

Reviewer #1: Yes

Reviewer #2: Yes

---

4. Is the manuscript presented in an intelligible fashion and written in standard English?

PLOS ONE does not copyedit accepted manuscripts, so the language in submitted articles must be clear, correct, and unambiguous. Any typographical or grammatical errors should be corrected at revision, so please note any specific errors here.

Reviewer #1: Yes

Reviewer #2: Yes

---

5. Review Comments to the Author

Please use the space provided to explain your answers to the questions above. You may also include additional comments for the author, including concerns about dual publication, research ethics, or publication ethics. (Please upload your review as an attachment if it exceeds 20,000 characters)

Reviewer #1: COX7A1-mediated mitochondrial dysfunction can induce ferroptosis in the endometrial

cancer cells

The study investigates the role of COX7A1 in mitochondrial dysfunction and ferroptosis in endometrial cancer. Bioinformatics analysis identified differentially expressed genes related to ferroptosis, and in vitro experiments confirmed that COX7A1 overexpression inhibits cancer cell proliferation and induces ferroptosis cell death. COX7A1 increased intracellular Fe2+ and MDA levels, reduced the GSH/GSSG ratio, and altered mitochondrial membrane potential. The findings of this study imply the significance of the COX7A1 gene as a driver of mitochondrial dysfunction and cellular stress in endometrial cancer cells. Although this gene has been investigated in other cancers, studying its role in endometrial cancer is also important. However, I have the following comments and concerns regarding the manuscript.

1) The abstract, conclusion, and discussion primarily focus on overexpression data obtained from Ishikawa cells. There should be a discussion on knockdown in AN3 CA cells and why this marker was highly expressed in these cells.

Response: Thank you for your valuable comments. We agree with your perspective. We have added the relevant changes in proteins and ferroptosis after AN3 CA knockdown in the abstract, conclusion, and discussion sections.

Main revisions in the abstract:

In vitro experiments have demonstrated that overexpression of COX7A1 inhibits the proliferation of endometrial cancer cells and induces ferroptosis by regulating intracellular iron metabolism and mitochondrial function. The specific mechanisms include increasing intracellular Fe^{2+} and malondialdehyde (MDA) levels, decreasing the GSH/GSSG ratio, and disrupting mitochondrial membrane potential, thereby leading to mitochondrial dysfunction. Furthermore, COX7A1 overexpression significantly reduces the expression of glutathione peroxidase 4 (GPX4) and SLC7A11, while upregulating acyl-coenzyme A synthetase long-chain family member 4 (ACSL4). In contrast, knockdown of COX7A1 promotes the proliferation of endometrial cancer cells and inhibits

ferroptosis, exhibiting the opposite effects. These findings provide new insights into the molecular mechanisms of endometrial cancer（Lines 39 - 49）.

Main revisions in the discussion:

Notably, overexpression of COX7A1 significantly reduced the expression levels of GPX4 and SLC7A11 while increasing the expression of ACSL4. Conversely, knockdown of COX7A1 resulted in upregulation of GPX4 and SLC7A11 and decreased ACSL4 levels. These observations are consistent with the classic ferroptosis mechanism, which induces cell death through lipid peroxidation and disruption of the antioxidant system （Lines 434-439）.

Main revisions in the conclusion:

In contrast, low expression of COX7A1 exhibits the opposite effect, promoting tumor cell proliferation. These findings not only reveal a novel mechanism by which COX7A1 inhibits the progression of endometrial cancer through the ferroptosis pathway but also suggest its potential as a therapeutic target for ferroptosis-based therapies（Lines 481-486）.

We observed that almost all endometrial cancer cell lines expressed COX7A1, except for Ishikawa cells, suggesting a potential regulatory role of this gene in these cancers, which warrants further discussion. The results based on Ishikawa cells cannot be generalized to other pancreatic cancer cells unless data are available showing the same result of the overexpression in the cells, which already expresses COX7A1.

Response: We completely agree with your opinion. To elaborate our viewpoints more clearly and respond to your questions, we are providing more detailed background and explanations here, hoping to dispel your concerns:

One of the core and consistent findings in our study is that the basal expression of COX7A1 can be detected in the vast majority of endometrial cancer cell lines we examined. Significantly, compared with normal cells (HEEpiC - SV40), the expression of COX7A1 is lower in endometrial cancer cell lines, and this difference is statistically significant (Figure 3A).

Our central hypothesis is that COX7A1 is a tumor suppressor with down - regulated expression in cancer, and the inactivation or reduced expression of its function is conducive to tumor cells evading ferroptosis.
Therefore, in Ishikawa cells, we demonstrated that "introducing" COX7A1 can inhibit tumor growth.
Conversely, knocking down the basal - expressed COX7A1 in these cells promotes tumor proliferation (as described in the original text).
These two sets of experimental evidence, one positive and one negative, form a complete logical loop under multiple cell backgrounds, jointly supporting the conclusion that COX7A1 acts as a broad - spectrum regulatory factor. Its biological effects do not depend on whether a specific cell line expresses it "from scratch", but rather on its relative expression level and functional state.
2) The absence of COX7A1 in Ishikawa cells suggests that the absence of this gene contributes to the invasive nature of these cells. In contrast, why is the COX7A1 gene highly expressed in AN3 CA cells? Does this indicate that these cancer cells are less invasive and aggressive?
Response: Thank you for raising such profound questions. You've astutely observed a key phenomenon in our data: COX7A1 is absent in Ishikawa cells but highly expressed in AN3 CA cells, and you've logically questioned its impact on cell invasiveness. This is indeed a point worthy of in - depth exploration.
First of all, we fully agree with your observation. The differences in COX7A1 expression levels among different cell lines are likely to reflect the heterogeneity of endometrial cancer and its different molecular subtypes. Our core explanation for this phenomenon is that the absolute expression level of a single gene is not always in a simple linear negative correlation with the final cell phenotype (such as invasiveness).
Despite the differences in absolute levels, our research has revealed a more crucial common pattern:
Compared with normal tissues: As you know, we have clarified in the paper that in endometrial cancer tissue samples, the expression of COX7A1 is generally

significantly lower than that in paired normal endometrial tissues. Even though the basal expression level of COX7A1 in AN3 CA cells is relatively high, our functional experimental data show that further knocking down COX7A1 in these cells can still significantly promote cell proliferation (Figure 4A). Conversely, overexpressing COX7A1 in Ishikawa cells can also lead to a further inhibition of cell proliferation.

However, the main focus of this study was on discussing the effects of COX7A1 on the proliferation, ferroptosis, and mitochondrial dysfunction of endometrial cancer. We acknowledge that the basal expression of COX7A1 may have varying degrees of impact on the invasion and migration of different endometrial cancer cells, but this still requires further investigation in our subsequent experiments. Once again, thank you for your valuable comments, which have given us a deeper understanding and insights into the research direction and cell line selection of this project.

3) Figure 6A: What is the quantified percent change in ROS? Please mention the percentage count of cells that expressed ROS.

Response: Thank you for raising this crucial question. We have re - composed the figures in terms of percentages as follows. We've noticed that in most papers, presenting the average fluorescence intensity of ROS and the positive ratio as output results are both quite common. If you need us to change it to the ROS positive ratio, please let us know. We will be happy to make the modification.

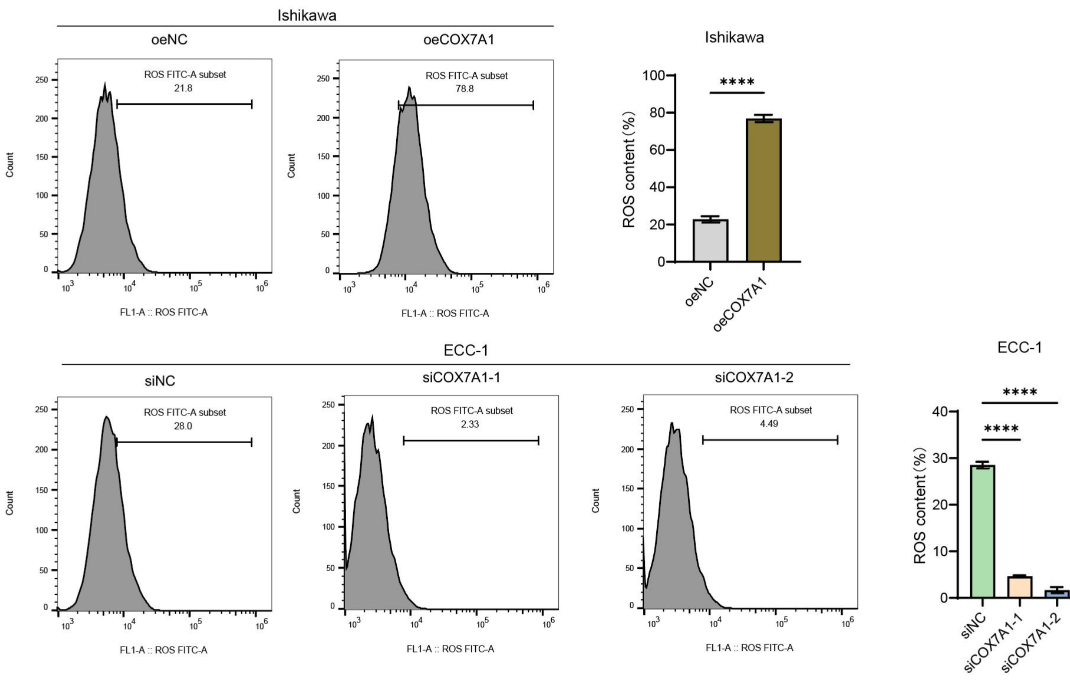

4) Figure 3 B: Data after transfection, the relative change was measured. Please add transfection efficiency data in the supplemental file. Additionally, please include the protein level expression of COX7A1 after transfection.

Response: We have added validation experiments related to transfection efficiency in the supplementary files, including experimental data from RT - qPCR and Western blot (the newly added experiment Figure 3C) for inspection.

5) What is the difference btw siCOx7A1-1 and siCOx7A1-2?

Response: Both siCOX7A1 and siCOX7A1 are different sequences of small interfering RNAs (siRNAs) targeting COX7A1. After transfection, they can specifically knock down the relative expression levels of COX7A1 mRNA and protein. The primer sequences are detailed in S1 Table.

What is your OENC and SiNC?

Response:OENC (Overexpression Empty Vector Negative Control)

Meaning: Overexpression empty vector negative control group.

Experimental procedure: In Ishikawa cells, a "blank" plasmid vector without the coding sequence of the COX7A1 gene was transfected.

Core objective: To rule out any non - specific effects on cells caused by the plasmid vector itself and the transfection process.

SiNC (siRNA Negative Control)

Meaning: Small interfering RNA negative control group.

Experimental operation: In AN3 CA cells, a non - targeted siRNA sequence that does not match the COX7A1 gene sequence was transfected.

Core purpose: To eliminate non - specific effects on cells caused by the siRNA molecule itself and the transfection process. The specific sequence information is detailed in Table S1.

We have added the supplementary information in lines 143-145 of the Methods section to make it easier for readers to understand our abbreviations. Thank you for your valuable comments, which will greatly improve the readability of our manuscript.

Did you select (transfected cell selection) the knockdown and overexpression cells and then use them for the downstream experiments?

Response:We sincerely appreciate the important question you raised. Regarding the screening of transfected cells, we fully agree that antibiotic selection is a crucial step in the construction of stable transfected cell lines, as you mentioned. In our experiment, for the sake of research objectives and timeliness, we adopted a strategy of transient transfection (including overexpression plasmids and siRNAs), and thus did not carry out antibiotic selection. This choice was based on the actual needs of the experimental design. We fully understand your concern in this regard and are more than willing to supplement relevant experimental details or conduct necessary verification if required. Thank you for your valuable comments, which are of great significance for us to improve our research work.

6) For your cck8 experiment, did you normalize your data against cell number and background absorbance?

Response: We are truly grateful for these professional and crucial questions you've raised. They have prompted us to think more deeply about the data analysis and interpretation of the CCK8 experiment.

In the data processing of this experiment, we did perform background subtraction, but we did not normalize the data at different time points based on the cell count. Our usual procedure is to subtract the OD value of the well containing only the culture medium and CCK8 reagent but no cells (blank control) from the OD value of each experimental group. This is to eliminate the influence of background absorption. For the data at different time points, we mainly directly compared the relative changes in the background - subtracted OD values of the treatment group and the control group at the same time point, so as to evaluate the impact of the treatment on cell viability. (Lines 156 - 160)

What does your OD data tell you about the cell state?

Response:The CCK8 reagent measures the activity of cellular dehydrogenase, which indeed mainly reflects the metabolic activity of cells. An increase in the OD value generally indicates an increase in the number of active and metabolically active cells. Under conditions where cells do not experience significant stress or toxic reactions, this enhancement of metabolic activity is positively correlated with the proliferation of cell numbers. Therefore, in the context of our study (evaluating the impact of gene manipulation on cell growth), the relative change in the OD value is used as a reliable indicator to measure cell proliferation/viability.

The reagent (tetrazolium salt) provides information about the metabolic state of cells, but did you perform cell counting to assess cell proliferation? If so, please mention the data.

Response:Your suggestion of directly evaluating proliferation by cell counting is an excellent validation method. In this study, we did not perform cell counting on parallel samples in the CCK8 experiment, which is a limitation in our experimental design. We mainly relied on the widely - accepted standardized CCK8 method to assess the proliferation trend. To address your concern, we have already stated the limitations of CCK8 as a detection method for proliferation experiments in the discussion section (lines 466 - 468).

7) After plating cells for EDU, how long did you incubate before adding the EDU solution? The fluorescence images just represent a selected part of the well. How did you quantify and count edu positive cells? Did you take images of all sites of the well, or did you perform flow cytometry to count the cells?

Response: We sincerely appreciate your valuable comments regarding the details of the EdU experiment. They have been instrumental in helping us present the experimental procedures more comprehensively and clearly. Below are our responses to your questions:

Regarding the incubation time from cell seeding to the addition of EdU:

In the experimental design, after cell seeding, the cells were treated according to the requirements of each group. After the treatment was completed, the EdU working solution was added and cultured for approximately 2 hours.

Regarding the representativeness of fluorescence images and the quantification method:

The issues you pointed out are of great significance. We used fluorescence microscopy for photography and quantification instead of flow cytometry. To ensure the representativeness of counting and reduce bias in field selection, we took the following measures:

Random multi - field collection: For each experimental sample (a single well in a 6 - well plate), we systematically and randomly selected at least 5 non - overlapping fields of view for photography. These fields of view evenly covered the central and peripheral areas of the well.

Software quantification process: We used Image J software to perform batch analysis on all the collected original images. The basic process is as follows: First, the total cell nuclei were identified by threshold segmentation using the DAPI channel image; then, the EdU - positive cell nuclei were identified by setting the same threshold using the EdU (usually Alexa Fluor 555) channel image; finally, the ratio of the number of EdU - positive cell nuclei to the total number of cell nuclei in each field of view was calculated (lines 173 - 175).

Regarding why flow cytometry was not used:

We fully agree that flow cytometry is one of the gold - standard methods for counting cell cycle or proliferation positive rates, as it can provide more objective and high - throughput single - cell data. In the preliminary phenotype screening of this study, we chose the microscopy - based EdU detection method because, while providing quantitative data, it can retain the spatial information of cell morphology and staining localization, facilitating us to visually verify the labeling efficiency. We acknowledge that supplementing flow cytometry data can further enhance the persuasiveness. We will consider this suggestion as a limitation of this study and use flow cytometry for verification in future in - depth mechanism research (lines 464 - 466).

We will add these explanations to the methods section of the revised manuscript.

Once again, thank you for your meticulous review. These comments have significantly improved the rigor of our work presentation.

8) Why did you choose to do the knockdown for COX7A1 in AN3 CA rather than overexpressing it? What purpose does the presence of COX7A1 in these cells serve? If we overexpress COX7A1 in AN3 CA rather than knock it down, will we experience the same inhibitory effects?

Response: We are truly grateful for this extremely crucial and in - depth question you raised. It has compelled us to more clearly elaborate on and reflect upon the scientific logic underlying the design of our experimental models. We fully understand your consideration that the symmetry and rigor of the experimental design are of vital importance for establishing the function of COX7A1. Here are our specific thoughts and answers to your queries:

Reasons for choosing knockdown instead of overexpression of COX7A1 in AN3 CA cells:

The core principle guiding our selection of experimental models is based on the endogenous basal expression levels of COX7A1 in different cell lines. Our aim is to construct two complementary models of "gain - of - function" and "loss - of - function" to more comprehensively validate its function. As shown in Figure 3A of the paper, among the various endometrial cancer cell lines screened, Ishikawa

cells have the lowest expression level of COX7A1, while AN3 CA cells have relatively the highest expression. Therefore, we performed overexpression in Ishikawa cells with low COX7A1 expression to simulate the possible effects of restored expression in tumors. Correspondingly, we carried out knockdown in AN3 CA cells with high COX7A1 expression to mimic the potential impacts of down - regulated expression. This design, based on endogenous expression differences, aims to be closer to the two directions of gene expression changes under physiological or pathological conditions, thus verifying the phenotypic effects of COX7A1 on cell proliferation and ferroptosis from two aspects. Our data indicate that knocking down COX7A1 in AN3 CA cells indeed promotes cell proliferation and inhibits ferroptosis markers, which is contrary to the effects of overexpressing COX7A1 in Ishikawa cells, where it inhibits proliferation and induces ferroptosis. These two opposite results corroborate each other, strengthening the conclusion that COX7A1 acts as an inhibitor of endometrial cancer cell proliferation and a driver of ferroptosis.

The basal function of COX7A1 in AN3 CA cells:

In untreated AN3 CA cells, the relatively high endogenous COX7A1 expression may be involved in maintaining the normal function of mitochondrial respiratory chain complex IV, thereby supporting basic cellular energy metabolism and redox balance. The results of our knockdown experiment suggest that when this endogenous expression is disrupted, the cell proliferation ability is enhanced, and the sensitivity to ferroptosis decreases. This indicates that in AN3 CA cells, the basal level of COX7A1 may play a role in inhibiting excessive proliferation and maintaining a certain sensitivity to ferroptosis. The specific mechanism may involve regulating the cell's metabolic state and oxidative stress level by modulating mitochondrial functions (such as membrane potential and energy metabolism), thus indirectly regulating the balance between proliferation and death.

Expected effects of overexpressing COX7A1 in AN3 CA cells:

This is an extremely insightful experimental suggestion. Based on the results that overexpressing COX7A1 in Ishikawa cells (with low endogenous expression) can significantly inhibit proliferation and induce ferroptosis, we reasonably speculate that further overexpression in AN3 CA cells, which originally have high COX7A1 expression, should theoretically produce inhibitory effects in the same direction but possibly to a different extent. However, we also recognize that there may be "threshold" or "saturation" phenomena in biological effects. That is, when the expression of COX7A1 exceeds a certain level, its effects may no longer increase linearly. In addition, differences in the genetic background and metabolic state of different cell lines may also affect the phenotype of overexpression. We fully understand that supplementing this experiment can perfectly form a complete verification cycle, making the conclusion more persuasive.

Due to the enormous workload of simultaneously supplementing overexpression and knockdown in different cell lines, we have chosen to conduct this experiment in future research. At the same time, we have explained the limitations of this choice in the discussion section (Lines 469-473).

Once again, we sincerely thank you for your rigorous and insightful review, which is of great value in improving the quality and depth of our research.

Minor comments :

1) Explain the abbreviations in figure legends like NC, siNC, oeNC, and all the markers you studied.

Response: We have added explanations of siNC, siCOX7A1 - 1, siCOX7A1 - 2, oeNC, and oeCOX7A1, as well as the full names of relevant markers in the figure legends according to your suggestion. This will make it easier for readers to understand our experimental groups.

2) Please improve the grammar of the abstract, e.g, Line 2 of the abstract should say "we explored.." and also line 8 of the abstract should read: 'Effect of COX7A1 on the inhibition of endometrial cancer cell growth.'

Response: We have made the modifications as per your suggestions. At the beginning of the second line in the abstract, it is now "we explored...". Also, the last sentence in the eighth line has been changed to 'Effect of COX7A1 on the inhibition of endometrial cancer cell growth.' Thank you very much for your valuable suggestions, which will significantly improve the quality of our manuscript.

Response: We have supplemented the number of repetitions for each experiment in the figure legend.

4) The ferroptosis and cell stress induction mechanism of COX7A1 can be observed in other cancers as well, such as lung and gastric cancers. Make sure you add relevant references in the manuscript. (https://www.nature.com/articles/s41419-022-05430-3, https://www.sciencedirect.com/science/article/pii/S0006291X16319659, https://eurjmedres.biomedcentral.com/articles/10.1186

Response: We have ensured that the above - mentioned research is presented in our manuscript. The link https://www.nature.com/articles/s41419 - 022 - 05430 - 3 is Reference 40 in the manuscript. The link https://www.sciencedirect.com/science/article/pii/S0006291X16319659 is the newly - added Reference 36, which is added in line 396 of the discussion section of the manuscript. The link https://eurjmedres.biomedcentral.com/articles/10.1186 could not be found during the page search, so it has not been added to this manuscript.

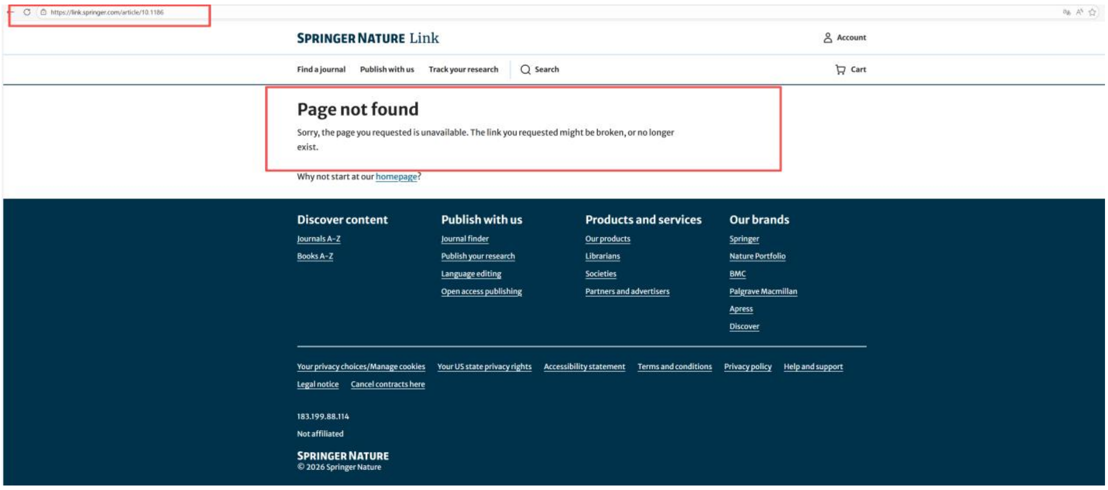

Reviewer #2: Comments to authors (s)

The research article presented by Qi Wu et al. on COX7A1-mediated mitochondrial dysfunction can induce ferroptosis in endometrial cancer cells has a minor issue with the manuscript.

The authors need to address the following comments.

•Please elaborate the material and methods section, like the double antibiotics name, trypsin solution dose, and full abbreviation of buffer, etc., for better understanding.

Response: According to your suggestions, we have provided supplementary explanations for the abbreviations used throughout the materials. The specific modifications are all in the "Methods" section, and the relevant changes are as follows:

Gene Expression Omnibus (GEO)

Principal Component Analysis (PCA)

Differentially Expressed Genes (DEGs)

Dulbecco's Modified Eagle Medium (DMEM)

1% (v/v) penicillin-streptomycin

phosphate-buffered saline (PBS)

oeNC (Overexpression Empty Vector Negative Control)

siNC (siRNA Negative Control)

4',6-diamidino-2-phenylindole (DAPI)

2',7'-dichlorodihydrofluorescein diacetate (DCFH-DA)

radioimmunoprecipitation assay (RIPA)

bicinchoninic acid (BCA)

polyvinylidene difluoride (PVDF)

Tris-buffered saline with 0.1% Tween-20 (TBST)

•Do the authors think that the COX7RP gene is also associated with ferroptosis in endometrial cancer, like COX7A1?

Response: We are truly grateful for your profound and insightful questions. Your attention to the potential functions of the COX7RP gene has provided us with a valuable direction for further improving the logical extension of our research and exploring the functional diversity of mitochondrial respiratory chain complex subunits in tumors. We sincerely appreciate this.

Based on the currently available research data, COX7RP has been confirmed as an oncogene in liver cancer (PMID: 32509385). However, its functional mechanisms are mainly related to mitochondrial metabolism and hypoxia adaptation (PMID: 35178385), and its direct association with ferroptosis has not been clearly reported.

Unlike COX7A1, which we discovered in our research to be a ferroptosis - driving factor and a tumor inhibitor, COX7A1 plays the role of a tumor - suppressor gene in endometrial cancer.

•How does the COX7A1 affect the cell metabolism in endometrial cancer?

Response: Thank you for this important question. Based on the data of this study, we believe that COX7A1 drives the remodeling of cell metabolism towards the conditions necessary for ferroptosis by inducing mitochondrial dysfunction. The specific impacts are summarized as follows:

The overexpression of COX7A1 leads to a significant decrease in the mitochondrial membrane potential (ΔΨm) (Figure 7A) and a down - regulation of the expression of ATP5A1, a key protein in energy metabolism (Figure 7B), suggesting that it impairs the normal energy - metabolic function of mitochondria.

The overexpression of COX7A1 causes a significant increase in intracellular reactive oxygen species (ROS) levels (Figure 6A). Meanwhile, it is accompanied by a down - regulation of the expression of GPX4, a core antioxidant enzyme, and a decrease in the GSH/GSSG ratio (Figure 5C, 6C), indicating that the cellular redox balance is disrupted and the antioxidant capacity is impaired.

The overexpression of COX7A1 results in an increase in intracellular Fe^{2+} levels (Figure 5A) and an elevation of malondialdehyde (MDA), the end - product of lipid peroxidation (Figure 5B). At the same time, the expression of ACSL4, a protein promoting ferroptosis, is up - regulated, while the expression of SLC7A11, which is involved in cystine uptake, is down - regulated (Figure 6C). These changes constitute the typical metabolic characteristics of ferroptosis. The morphological changes such as mitochondrial shrinkage observed by electron microscopy (Figure 6B) are consistent with these findings.

The results of this study indicate that in endometrial cancer cells, overexpression of COX7A1 may induce lipid peroxidation and ferroptosis by impairing mitochondrial function, triggering ROS accumulation and redox imbalance, and simultaneously altering the expression of proteins related to iron and lipid metabolism.

It should be noted that this study has verified the changes in these key metabolic nodes. However, some of the upstream - downstream molecular relationships

among these nodes still need to be further clarified in future research. We have incorporated the above content into the discussion section (lines 415 - 431).

Thank you for your valuable comments, which are of great significance for us to improve the presentation of our research.

•Why do the authors think that the microenvironment of different cancers can regulate the COX7A1 and how?

Response: We are truly grateful for this profound and crucial question. Our proposition that "different cancer microenvironments may differentially regulate COX7A1" is mainly based on the following two observations and lines of reasoning:

Firstly, the most straightforward evidence is the contradictory functions of COX7A1 in different cancers. For instance, it has been reported as a tumor - suppressor gene in non - small - cell lung cancer, capable of inducing cell death [35, 36]. In contrast, in gastric adenocarcinoma, its high expression is associated with poor prognosis and chemoresistance [37, 38]. This strong "tissue/environment - dependence" implies that its biological effects are not inherent but are shaped by the specific tumor microenvironment (TME) in which it is located.

Secondly, regarding the possible regulatory mechanisms of the microenvironment: 1) Epigenetic regulation: Microenvironmental stresses such as hypoxia and inflammation may regulate the expression of COX7A1 at the transcriptional level through mechanisms like methylation. 2) Functional context remodeling: More importantly, the TME may determine whether COX7A1 activates the "pro - death" or "pro - survival" downstream network by altering the cellular metabolic state, immune cell infiltration (such as the activation of cancer - associated fibroblasts), or the signaling pathway context. Of course, these ideas are speculations based on our current research findings combined with the literature. There is no direct evidence to verify the relationship between COX7A1 and the tumor microenvironment. Therefore, we did not incorporate

this speculation into the manuscript. If you require this part to be included in the manuscript, we will be happy to make the second - round revisions.

•Do the authors think monitoring other lipid peroxidation products would also be beneficial?

Response: We are sincerely grateful for this valuable and professional suggestion. We fully concur with your view that monitoring other lipid peroxidation products is of great significance for a more comprehensive clarification of the lipid metabolism basis of ferroptosis.

In this research, we selected malondialdehyde (MDA) as the core detection index mainly because it is one of the most classic and stable end - products of lipid peroxidation. Its level can reliably reflect the overall degree of lipid peroxidation and has been extensively used as a key biochemical marker for ferroptosis. Our data indicate that the overexpression of COX7A1 can significantly increase the MDA level, providing solid evidence for the occurrence of lipid peroxidation.

Nevertheless, we are well - aware that lipid peroxidation is a complex cascade reaction. Monitoring other products, such as:

Early/primary products: Lipid hydroperoxides, for example, can more directly reflect the onset of peroxidation.

Bioactive secondary products: 4 - Hydroxy - 2 - nonenal not only serves as a damage marker but also functions as a signaling molecule involved in regulating cellular responses.

Oxidation products of specific phospholipids: Oxidized phosphatidylethanolamine, for example, is a more specific mediator in ferroptosis.

Monitoring these products will assist in differentiating the stages of peroxidation, revealing more specific lipid substrates, and exploring the signaling functions of oxidized lipids, thereby advancing the mechanistic research to a more refined level.

We will incorporate this profound insight into the paper revision. We plan to add relevant content in the discussion section, acknowledging the limitations of this study in terms of detecting lipid peroxidation products, and designating "systematically analyzing different lipid peroxidation intermediates and end - products using techniques like lipidomics" as an important future research direction (lines 471 - 473).

Your suggestion has significantly enhanced our understanding of this issue. Thank you again wholeheartedly.

---

6. PLOS authors have the option to publish the peer review history of their article (what does this mean?). If published, this will include your full peer review and any attached files.

If you choose "no", your identity will remain anonymous but your review may still be made public.

Do you want your identity to be public for this peer review? For information about this choice, including consent withdrawal, please see our Privacy Policy.

Reviewer #1: **Yes:** Sonia Kiran

Reviewer #2: **Yes:** ABHIRUCHI KANT

[NOTE: If reviewer comments were submitted as an attachment file, they will be attached to this email and accessible via the submission site. Please log into your account, locate the manuscript record, and check for the action link "View Attachments". If this link does not appear, there are no attachment files.]

To ensure your figures meet our technical requirements, please review our figure guidelines: https://journals.plos.org/plosone/s/figures
You may also use PLOS's free figure tool, NAAS, to help you prepare publication quality figures: https://journals.plos.org/plosone/s/figures#loc-tools-for-figure-preparation.
NAAS will assess whether your figures meet our technical requirements by comparing each figure against our figure specifications.
